# Characterization of Stormwater Runoff Based on Microbial Source Tracking Methods

**DOI:** 10.3389/fmicb.2021.674047

**Published:** 2021-06-10

**Authors:** Silvia Monteiro, Gaspar Queiroz, Filipa Ferreira, Ricardo Santos

**Affiliations:** ^1^Laboratório Análises, Tecnico Lisboa, University of Lisbon, Lisbon, Portugal; ^2^Department of Civil Engineering, Tecnico Lisboa, University of Lisbon, Lisbon, Portugal

**Keywords:** stormwater, fecal contamination, mitochondrial DNA, microbial source tracking, urban environment

## Abstract

Rainfall and associated urban runoff have been linked to an increased deterioration of environmental waters, carrying several pollutants including pathogenic microorganisms. Such happens because fecal matter is washed into storm drainage pipes that are afterward released into environmental waters. Stormwater has not been extensively characterized as it is, because most studies are performed either on drainage pipes that are often impacted by sewage leakage or directly in environmental waters following a rain event. In this study, stormwater collected directly from the streets, was monitored for the presence of fecal indicator bacteria (FIB) and three potential important sources of fecal contamination in urban environments (human, cats, and dogs) in three distinct basins in Lisbon, Portugal. Stormwater was collected in sterilized plastic boxes inserted in the storm drains, therefore collecting only runoff. High concentration of fecal contamination was detected with a high percentage of the samples displayed at least one source of contamination. A strong relationship was found between the number of detected sources and the precipitation levels. Although no statistical correlation was found between the locations and the presence of FIB or source markers, the results show a trend in geographical information on the type of urban use in each basin. To the best of our knowledge, this is the first study analyzing the runoff collected directly from the streets. This study suggests that, in urban areas, stormwater runoff is highly impacted by fecal matter, not only from domestic animals but also from human origin, before any cross-contamination in the drainage system and may, by itself, pose a high risk to human health and the environment, particularly if water reuse of this water without further disinfection treatment is the final goal.

## Introduction

In the last quarter of the twentieth century, most countries developed an increasing concern over urban pollution, trying to limit the impacts on natural environments of both solid waste and wastewater.

The preservation of receiving waters depends strongly on the quality of wastewater and stormwater discharges. The latter is a direct result of precipitation over an urban basin, dragging all pollutants accumulated during dry weather: rooftop and traffic pollutants, single discharges of waste and pollutants from several other sources. A number of different studies have found that pollutant loads are strongly related to factors of three different natures: geomorphology of the catchment (Butler and Memon, 1999) local climacteric conditions or precipitation regime over the basin (Gnecco et al., 2005); and the type and intensity of land use (Gray, 2004).

Wastewater discharges are, for instance, being monitored and controlled in the European Union countries and in the United States, under international legislation – European Union Directive 2006/7/EC (CEC Council Directive, 2006) and U.S. Clean Water Act 1972 (USEPA, 1972) – which tries to limit the impact on natural environments by these waters. Despite the growing concerns in the preservation of receiving waters, and the increasing demand in water quality standards, there is no legislation concerning stormwater in particular, and no mandatory control over stormwater runoff. Moreover, there is a growing need to find alternative water sources, especially in areas characterized by water scarcity. About four million people already life in locations with high water stress at least 1 month per year (Mekonnen and Arjen, 2016) and it is expected that approximately, one-third of the world’s population will be impacted by physical and/or economic water scarcity by the year 2025 (UNCCD, 2014). Several water sources have been proposed, including stormwater since it is considered a relatively clean, and affordable alternative water source. A large number of studies have shown the presence of pathogenic organisms in stormwater (Ahmed et al., 2010, 2011, 2014; Jongman and Korsten, 2016; Waso et al., 2016; Bae et al., 2019).

Current fecal indicator bacteria (FIB) do not allow allocating the source of detected fecal contamination. Tracking the source of microorganisms allows for a better understanding of the water cycle in general and urban stormwater behavior in particular. Methods trying to identify the source of fecal contamination are called source tracking (ST) methods. Most ST methods are based on the premise that different intestinal systems select for different microorganism populations, due to diet and digestive differences of their hosts (Santo Domingo et al., 2007). There are, however, different approaches like the use of species-associated eukaryotic mitochondrial DNA markers (mtDNA) for the direct determination of sources of contamination.

Several studies tested a great variety of genetic markers, trying to assert the source of a given fecal contamination (Ballesté et al., 2010) in several different countries and hydrological contexts, namely: in rural hydrological catchments in Austria (Reischer et al., 2011) and California, United States (Kildare et al., 2007) in rivers in Canada (Martellini et al., 2005; Kortbaoui et al., 2009) and Spain (Ballesté and Blanch, 2010); and in surface and coastal waters of the United Kingdom (Baker-Austin et al., 2010). These methods have also been applied to more urban contexts to identify human fecal contamination in natural water courses during wet weather, in Australia (Ahmed et al., 2008), and to demonstrate the existence of illegal connections into stormwater collectors, that continuously shed a steady stream of human fecal contamination, even in dry weather periods, in California, United States (Sercu et al., 2009).

In this study, mtDNA markers were selected because most ST approaches used were not completely reliable (USEPA, 2005) and do not provide solutions for the discrimination of some of the animals targeted in this study. Therefore, the aim of this study was: (i) design and validate mtDNA markers targeting domestic animal fecal contamination (cat and dog); and (ii) analyze stormwater collected directly from the streets following rain events, for the presence of FIB and MST markers targeting human, dog, and cat fecal contamination. By using mtDNA markers, the nucleic acid of the animal species is targeted directly instead of microbial-associated species that may vary due to dietary and climate differences.

To the best of our knowledge, this is the first study conducted in the runoff collected directly from the streets, following a rain event seeking to: (i) evaluate the quality of stormwater runoff in the city of Lisbon, with special focus on fecal contamination levels; (ii) use mtDNA markers designed specifically for species commonly found in the urban environment (humans, cats and dogs) to assess the origin of registered fecal pollution in the city of Lisbon; and (iii) correlate the sources of pollution with the activity areas within the city of Lisbon. Such study will allow for a better understanding not only of the levels of fecal contamination of these waters but will also provide information on the potential sources and ultimately, the microbiological risks of discharging such waters directly in the environment.

## Materials and Methods

### Experimental Basins

Three experimental basins were chosen in Lisbon city center due to their quite dissimilar topographic, morphologic and urban use characteristics: Alcântara (A), Bairro das Ilhas (I) and Madalena Street (M) (Supplementary Figure 1). Alcântara basin has residential areas as well as areas more dedicated to commerce and nightlife. There are streets with intense traffic, and both steep and flat streets. Most streets have trees in the sidewalk, intensifying the presence of vegetal debris. The basin Bairro das Ilhas is significantly smaller than Alcântara, and is mainly residential with no commercial activity, tight one-way streets and the average slope is generally smooth. The traffic is of low intensity with areas of exclusive pedestrian access, with only one relevant green space with 4,000 m^2^. We also observed that some of the buildings’ rooftops drain directly to the pavement. The third experimental basin include Madalena Street, right in the middle of the 18th century historical center of Lisbon. The street has an intense commercial activity and many residential areas. There are streets with intense traffic, with bus lines and tramlines.

### Rain and Temperature Data

Rain data were supplied by Instituto Geofísico Infante Dom Luiz (IGIDL) and by Laboratório Nacional de Engenharia Civil (LNEC). Results obtained of instantaneous precipitation for 2011 and 2012, in each of the rain gages (LNEC-NEC, LNEC-WWTP and IGIDL) are shown in Supplementary Figure 1, in Supplementary Material. Also shown in the graphics are the dates of the campaigns. The LNEC-WWTP rain gage did not register any data from 04-11-2011 around 11 a.m. to 28-11-2011 around 10 a.m., possibly due to a malfunction in the equipment. The differences between recorded rain were mainly due to relevant spatial variability.

Mean temperatures varied between 20∘C on October 2011 and 8∘C on February 2012 (Supplementary Table 1 in Supplementary Material) (IPMA, 2012).

### Sample Collection

**FIGURE 1:**
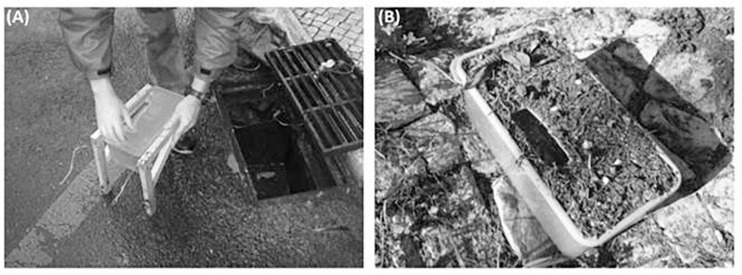
**(A)** Simulation of experimental design with the placing of sample boxes in storm drains. **(B)** Sediment deposition after a rainfall event.

The collection campaigns took place from October 2011 to May 2012. Immediately before each rain event, a previously decontaminated plastic box was inserted into the storm drains, where stormwater runoff was collected during that specific rain event (Figure 1). A total of 75 samples were collected during five rain events: six locations at Alcântara, six at Ilhas and three at Madalena per sampling event. The contents of the boxes were transferred to a sterile 1L sampling bottles (Deltalab, Spain), and taken to the laboratory at (5 ± 3) ∘C within 2 h of collection. Samples were processed within 8 h of collection for FIB and mtDNA markers.

### Fecal and Raw Wastewater Sample Collection

Fecal and raw wastewater samples were used for the validation of the newly designed domestic animals mtDNA markers (dog and cat). Fecal samples were collected using sterile tools and placed in sterile 50-ml tubes. Cow (10 samples), pig (9 samples), poultry (7 samples), pigeon (12 samples), gull (10 samples), rat (9 samples), cat (15 samples), and dog (15 samples) fecal matter was collected from different locations in the Lisbon area or donated by a variety of people and stores. Human fecal matter (15 samples) were provided by a variety of individuals of different ethnicities and age. All samples from individual hosts were transported to the laboratory at (5 ± 3) ∘C. Raw wastewater (20 samples) was collected at distinct wastewater treatment plants (WWTP) in Portugal and send to the laboratory refrigerated at (5 ± 3) ∘C. Upon arrival to the laboratory, all samples (fecal matter and raw wastewater) were immediately stored at (−30 ± 5) ∘C until further processing.

### Microbial Indicators

*Escherichia coli* (EC) and intestinal Enterococci (IE) were analyzed using Colilert and Enterolert (IDEXX, United States), respectively, according to the manufacturers’ instructions.

### Sample Concentration and DNA Extraction

One-liter of stormwater was centrifuged at 9,000 × *g* for 15 min (Sigma 3k-18, Sigma Germany) and the pellet resuspended in 5 mL of the supernatant. Mitochondrial DNA was extracted from 200 μL of concentrate using the QIAamp DNA Mini Kit (Qiagen, United States), following the manufacturer’s instructions. DNA elution was performed in 200 μL final volume. A process control, consisting of sterile distilled water, was analyzed with each experiment. In addition, an extraction control, consisting of nuclease-free water, was also conducted.

Fecal matter (220 mg) from the different animals and raw wastewater samples (220 μL) were extracted using the QIAamp DNA Stool Mini kit (Qiagen, United States), according to the manufacturer’s instructions, with DNA eluted in 200 μL final volume. Extracted samples were stored at (−30 ± 5) ∘C until further processing.

### Design of Primers for Detection of Fecal Contamination From Cat and Dog Origin

Specific primers were designed for the detection of fecal contamination from cat and dog origin. Human, cat, and dog mitochondrial DNA (GenBank database accession no. J01415, NC_028310, and KF907307, respectively) were aligned using the ClustalW software (European Bioinformatics Institute, United Kingdom). After the alignment, locations in the cat and dog mtDNA with the greatest divergence were chosen and inserted in the Primer Express^®^ 3 software (Applied Biosystems, United States) to obtain candidate primers. The chosen candidates were further analyzed, *in silico*, using BLAST to detect and eliminate those with interspecies reactivity (Altschul et al., 1990). Each set of primer pairs was analyzed together and separately to contemplate their use. For each species, a specific-primer pair was selected (Table 1) and used for nested PCR.

**TABLE 1:** List of primers used in PCR and nested PCR in this study.

**Source**	**Primers**	**Expected size**	**Reference**
Human			Martellini et al., 2005
**Single PCR**			
Humito2-G	5′AGCCCTTCTAAACGCTAATCCAAGCCT-3′	659	
Humito2-D	5′-CTTGTCAGGGAGGTAGCGATGAGA-3′		
Nested PCR			
Humito11-G	5′-CCACTACTAGGCCTCCTCCTA-3′	612	
Humito11-D	5′-TAGCGATGAGAGTAATAGATAGGG-3′		
Cat			This study
**Single PCR**			
CatMito1-F	5′-CCTGTCCACACTACTTGTACTCATCGC-3′	539	
CatMito1-R	5′-AGATGGTTGTTTAGGATGGCTACG-3′		
Nested PCR			
CatMito2-F	5′-ATTTGATCCTATAGGGTCCGCC-3′	350	
CatMito2-R	5′-CCTATGAGCGACATGATGAAAGC-3′		
Dog			This study
**Single PCR**			
DogMito1-F	5′-ATGGCTCTAGCCGTTCGATTAAC-3′	638	
DogMito1-R	5′-GGCTAGGAGGACTGAGGTGTTGAG-3′		
Nested PCR			
DogMito2-F	5′-CATTAGGATTCACAACCAACCTGTTA-3	236	
DogMito2-R	5′-AATAATGCCGGTAGGAGGTCAG-3′		

### Single and Nested PCR Conditions

Single PCR was performed in a 25 μL final volume reaction using 0.4 pmol/μL of each primer, 5 μL of extracted DNA using the illustra^TM^ puReTaq Ready-To-Go PCR Beads (GE HealthCare, United Kingdom) following the manufacturer’s instructions. Nested PCR was performed in the same conditions except that 1 μL of the single PCR reaction was used as template DNA and the internal primers were used. With each PCR, 10-fold dilution of every DNA extract was also evaluated. Process and extraction controls and positive and negative controls were analyzed with each PCR run. Amplifications were carried out in a Veriti 96 well thermal cycler (Applied Biosciences, United States) using the following conditions: (i) human: 94∘C for 5 min, 55∘C for 5 min, 35 cycles of 72∘C for 2 min, 94∘C for 40 s and 55∘C for 1 min, with a final elongation at 72∘C for 10 min (Martellini et al., 2005); and (ii) cat and dog: 94∘C for 5 min, 59∘C for 5 min, 35 cycles of 72∘C for 2 min, 94∘C for 40 s and 59∘C for 1 min, with a final elongation at 72∘C for 10 min. Amplicons were visualized after electrophoresis on 2.5% agarose gels.

### Validation of Primers

The validation of the assays consisted of two steps: (i) determining the sensitivity; and (ii) determining the specificity. First, targeted single-component fecal suspensions were analyzed by with the two designed assays in a nested PCR to test and determine the sensitivity. Sensitivity was determined by analyzing 10-fold dilutions of DNA, extracted from the feces of each animal and quantified using NanoDrop (Thermo Fischer Scientific). The lowest quantity detected in three separate experiments was chosen and determined as the limit of detection (LoD; sensitivity) for each assay. Secondly, each assay was surveyed for possible cross-reactivity (specificity) with other species such as human, cow, pig, poultry, pigeon, gulls, and rats. The cross-reactivity assay consisted of the analysis of mixed fecal suspensions composed of the non-target species mentioned above using the assays developed.

### Statistical Analysis

All data analysis was done either with Microsoft Excel 2016 or IBM SPSS Statistics 25 (IBM, United States). Data were converted into a logarithmic format. Three factors – sampling area, FIB concentration and precipitation levels – were analyzed for significant difference in the variance of the occurrence of each marker in the runoff. One-way ANOVA was conducted to determine the association between sampling location and the presence of FIB (EC and IE) and MST markers (Human, dog, and cat). The association between the presence of FIB and MST markers was assessed using One-way ANOVA. Spearman rank order correlation was used for calculation of correlation coefficients between FIB and precipitation levels.

## Results

### Microbial Indicators

**FIGURE 2:**
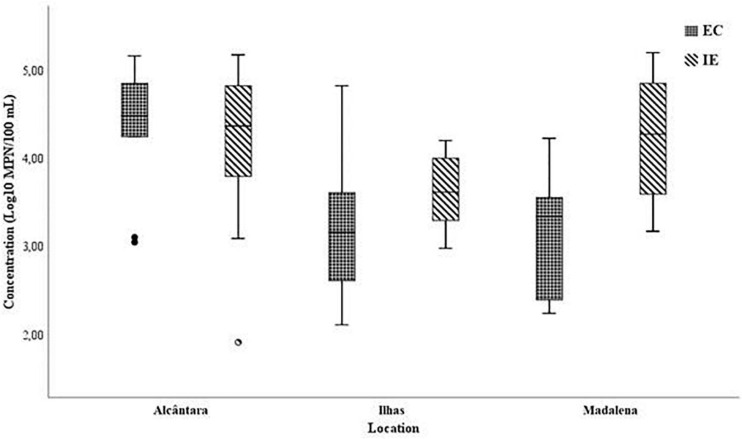
Concentration of EC (A) and IE (B) in the three locations chosen, Boxes, 25th and 75th percentile; Whiskers, 10th and 90th percentile; inside the boxes, median.

The percentage of positive samples for the three basins was 100% for IE and 95% EC (results for EC ranging from 100% positive samples in Ilhas and Madalena basins and 89% in Alcântara). The distribution of EC and IE concentrations for the three basins is presented in Figure 2. The highest EC concentration was found in Alcântara (mean concentration: 4.29 ± 0.66 log MPN/100 mL) followed by Madalena (mean concentration: 3.38 ± 0.92 log MPN/100 mL) and Ilhas (mean concentration: 3.22 ± 0.71 log MPN/100 mL). Concentration of IE was highest in Madalena (mean concentration: 4.28 ± 0.78 log MPN/100 mL), followed by Alcântara and Ilhas (mean concentrations: 4.07 ± 0.92 log MPN/100 mL and 3.63 ± 0.43 log MPN/100 mL, respectively). The concentration of IE was greater than that of EC in two of the basins, Ilhas and Madalena. For Alcântara, the concentration of EC was higher than the concentration of IE, although being undetected in some of the samples.

Crossing the results for EC and IE with the rain data, there is no perceivable impact on the concentration of IE due to an increase in precipitation (Table 2). For EC, only for the Ilhas basin, a negative impact with the precipitation intensity was observed (*r* = −0.528, *ρ* < 0.05; Table 2).

**TABLE 2:** Spearman rank order correlation between the levels of precipitation and the concentration of FIB per basin area.

	**Microorganism (spearman rank order correlation, *r*)**	
**Location**	**EC**	**IE**
Alcântara	0.114	0.146
Ilhas	−0.528	−0.297
Madalena	0.103	0.128

**FIGURE 3:**
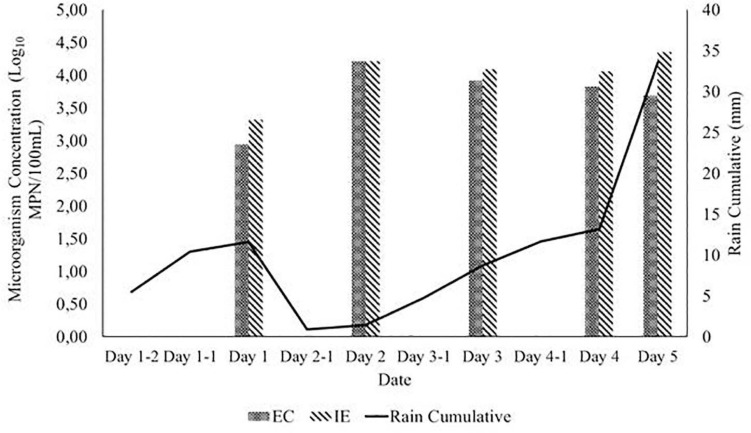
Mean concentration of EC and IE per rain event: Day X-2 represents the rain intensity.

Due to the impact of cumulative rain in the previous days of sampling, the mean concentration of EC and IE was calculated and compared with the cumulative values of rain in the day of the sampling and on the two previous days (Figure 3).

The mean concentration for both microorganisms was mostly similar throughout the sampling campaign showing little to no effect of the levels of rain in the number of microorganisms, with EC displaying generally lower mean concentration than IE (exception of sampling date 2). It is also important to note that no significant statistical difference was found for the presence of EC or IE with the location settings (One-way Anova, *p* > 0.05).

### Validation of Designed Primers

To determine the specificity of the designed primers for cat and dog sources, the primers were tested against fecal matter from different species, including humans, cow, pig, poultry, pigeons and gulls. These animals were chosen specifically since they could be the main sources of fecal contamination in urban and river environments found in the River Tagus catchment.

No cross-reactivity was observed for each species-specific primer, with the exception of a single raw wastewater sample that showed positive results for the dog targeting assay (Table 3). The assays developed for the specific detection of contamination from domestic animals produced positive results only when the corresponding species was analyzed representing the absence of non-specific amplifications in the fecal samples. The results are shown in Supplementary Figure 2.

**TABLE 3:** Specificity of the cat and dog mtDNA markers in samples from varying fecal origin.

**Source**	**Percentage of positive samples with targeted assays (no. positive samples/total no. of tested samples)**	
**Fecal matter**	**CatMito**	**DogMito**

Cat	100% (15/15)	0% (0/15)
Dog	0% (0/15)	100% (15/15)
Human	0% (0/15)	0% (0/15)
Cow	0% (0/10)	0% (0/10)
Pig	0% (0/9)	0% (0/9)
Poultry	0% (0/7)	0% (0/7)
Pigeon	0% (0/12)	0% (0/12)
Gulls	0% (0/10)	0% (0/10)
Rats	0% (0/9)	0% (0/9)
Raw Wastewater	0% (0/20)	5% (1/20)

Sensitivity tests were made for each set of nested-PCR by decreasing the quantity of the corresponding targeted animal DNA in the PCR reaction (Supplementary Figure 3). The sensitivity limit was determined to be around 1 pg for cat mitochondrial marker and 0.01 pg for dog mitochondrial marker following nested PCR.

### Evaluation of Sources of Fecal Contamination in the Catchment Area

**FIGURE 4:**
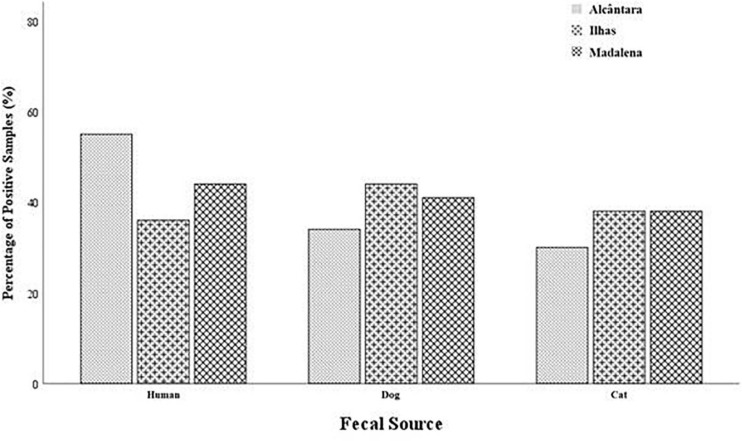
Percentage of positive samples in each basin per source of fecal contamination.

To determine the origin of fecal contamination responsible for the high concentration of EC and IE in the basins, three sources were evaluated due to their potential impact and presence in urban environments: human, cats and dogs. Data showed that 68% of the samples were positive for at least one of the targeted sources, with 9% showing positive signals for all sources tested. Human was the most relevant source, followed by dog and cat at 49, 38, and 35%, respectively. Figure 4 shows the percentage of positive samples for the three basins by source.

The prevalence of fecal markers in Alcântara was 55, 34, and 30%, for human, dog, and cat origin, respectively. In the remaining two basins, the prevalence of each marker was similar and closer. In Ilhas, the prevalence was 44, 41, and 36% for dog, cat and human assays, respectively. Finally, in the Madalena basin the results were 44, 38, and 38% for human, dog, and cat fecal contamination, respectively. In spite of the higher percentage of positive samples in Alcântara for human fecal contamination marker, there was no statistical significant difference between location and the presence of a particular fecal contamination (One-way Anova, *p* > 0.05). Additionally, no statistically significant difference was found when analyzing each fecal indicator bacteria (FIB) and the presence of targeted marker (One-way Anova, *p* > 0.05).

**FIGURE 5:**
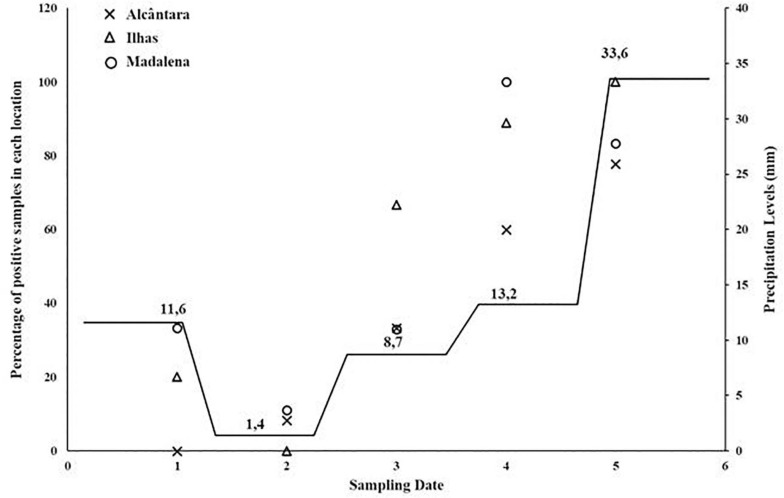
Relation between the levels of precipitation and the percentage of detected MST assays per sampling date.

There was statistically significant difference between the precipitation levels and the assays targeting dog and cat fecal contamination (*p* = 8.1 × 10^–15^, and *p* = 6.0 × 10^–6^, respectively; *p* < 0.001). Additionally, there was strong association between the number of fecal contamination targets detected (zero markers, one marker, two markers, and all markers detected) and the levels of fecal contamination (*p* = 1.1 × 10^–10^; *p* < 0.001). Sampling dates with greater precipitation levels also showed a larger number of detected sources whereas days with low precipitation levels had lower numbers of detected markers. Comparison between the presence of different fecal contamination markers and precipitation is showed in Figure 5.

Observing data from Figure 5 it is noticeable that sampling days with higher levels of precipitation (days 5 and 6) also displayed higher number of combined detected MST targeting assays, with detection of at least one MST marker in these days. For sampling date 2, displaying the lowest precipitation intensity, the combined number of MST markers varied between 0 and 1, at a mean marker detection level of 0.25. The highest percentage of positive samples was obtained during the month of May (80%) whereas the lowest was determined for the months of March and April (28 and 21%, respectively).

## Discussion

Little or no impact of the precipitation intensity on the concentration of FIB was detected throughout this study, with the exception of a moderate negative correlation between Ilhas basin and EC concentration. Such result may be a consequence of a “first flush” event caused by a possible increase in the levels of EC followed by dilution effect with continuing rain and from the fact that Ilhas is a smaller residential area, with no commercial or nightlife areas. Similar results have been previously shown, where different sampling locations also showed different correlation for EC during rainfall events (Kleinheinz et al., 2009). Billian et al. (2018) studied the effect of precipitation in the movement, and therefore in the levels of FIB, through septic drain fields and found no relationship between precipitation and FIB.

To determine the sources responsible for to the high levels of fecal contamination found in the runoff, mtDNA markers were chosen to contemplate three major potential sources in urban areas: human and domestic animals (dogs and cats). Data from this study has shown that the existence of a given source of contamination is not related to the presence of FIB. Moreover, EC and IE were detected in samples in the absence of the targeted sources of fecal contamination. Such results are possibly due to FIB inputs from other sources in urban areas such as pigeons and gulls. These results are in accordance with previous studies (Waso et al., 2018a; Hajj-Mohamad et al., 2019) that tested the use of different markers, including human mitochondrial DNA marker, to determine correlation between MST markers, FIB and land use and have determined that no correlation existed between the markers and EC in residential areas. A study by Staley et al. (2016) determined that human and gull were the most important sources of fecal contamination in stormwater outfalls, indicating a large input from such sources in the levels of contamination (Staley et al., 2016). Similarly, a study by Waso et al. (2018b) concluded that fecal contamination from pigeon origin had a high impact on the quality of harvested rainwater.

In the present study, although no direct statistical correlation was found between location and the presence of a certain source of fecal contamination, data provided trends on the different activities occurring in the chosen basins. The city of Lisbon does not have open sewers, therefore the mtDNA results can be directly imputed solely to urban runoff. Alcântara basin is a residential area with dense commercial areas, heavy traffic and packed with nightlife clubs, resulting in the highest percentage of positive results for human-associated marker and the lowest for domestic animals. Alcântara basin, with a high number of homeless individuals and with a vibrant nightlife is the area where the impact of mtDNA is more noticeable. Ilhas basin is a small and quiet residential neighborhood in Lisbon, where most of the households have their own private garage minimizing the time that the residents are on the sidewalks. Usually the domestic animals are taken for walks in the surrounding streets. This impact is markedly visible due to the lowest human fecal contamination and the highest contamination from domestic animals detected. On the other hand, data obtained for the Madalena basin is a reflection of the urban use of this area, a mix between Alcântara and Ilhas basin with a mix of residential and commercial areas. Interestingly, the concentration of EC was also higher than that of IE in Alcântara, but lower for the remaining chosen sites. The relation between these two indicators is commonly accepted as an indication of human/animal source identification, further corroborating the results obtained by this study using mtDNA.

Data for MST markers are in agreement with previously published work showing an increase in the detection of FIB and other organisms from fecal contamination following a precipitation event (Lipp et al., 2001; Kelsey et al., 2004; Mallin et al., 2009). Billian et al. (2018) have shown a correlation between precipitation and the presence of *Bacteroides* HF183 marker when analyzing the movement of MST markers and conventional fecal indicators through septic drain fields. Human and animal *Bacteroides* markers have been consistently identified as the “gold standard” in determining the sources of fecal contamination (Harwood et al., 2014). However, they present several challenges, including dietary and climate dependence which may diminish the sensitivity of these markers. The choice of targeting mtDNA for source tracking in this particular study resulted from several premises: (i) mtDNA markers target specifically eukaryote cell DNA from the target animals rather than targeting specific bacteria or viruses associated to a specific animal; (ii) mtDNA evolves faster than nuclear DNA therefore providing an adequate number of sequence variations for the design of species-associated PCR assays; (iii) a large number of exfoliated cells are released in the feces of animals each containing a high number of mitochondrion and respective DNA sequences, which increases largely the sensitivity of the assays; (iv) specific strains of *Bacteroides* spp. may not be present in all of their specific hosts, as is the case for instance of the HF183 marker which is not present in the intestinal microbiome of all humans (Harwood et al., 2009); and (v) *Bacteroides* spp. are obligatory anaerobes and in our particular setting this could conduct rather immediately to their lysis and subsequent DNA degradation. In addition, the HF183 marker has been detected in gull waste samples, not only from the feces of gulls but also in the cloacae, indicating that gulls may transport human fecal pollution to places not impacted directly by human action (Alm et al., 2018).

The results from this study highlight the need for a better control of urban runoff, that may be a carrier of pathogenic organisms with the potential to cause risk to human health, indicating also the extreme importance of sources of fecal contamination other than human and domestic animals in the quality of the runoff.

## Conclusion

Due to the experimental design, the present study, shows the direct analysis of urban runoff and the potential effects of this phenomenon in environmental waters. The majority of the studies targeting stormwater quality have been conducted either in the drainage pipes, many times cross-contaminated with sewage, or in environmental waters following a rain event. Such sampling approach change the load and type of fecal contamination that is being determined possibly masking largely the real input of urban runoff on the environment. This study shows, therefore, that stormwater runoff is one of the most important sources of biological pollutants that can potentially impair water quality and pose risks to the ecosystem and human health. Additionally, urban stormwater is being used in multiple situations to increase non-potable and potable water supplies within cities and other urban areas (Sidhu et al., 2012), with the potential to pose serious health problems. Based on the data presented in this study, it is important to identify the sources of fecal contamination in stormwater runoff for a better and more targeted remediation action to reduce risks to public health. In addition, disinfection treatment of stormwater must be performed if reuse is considered, even for non-potable purposes.

## Data Availability Statement

The original contributions presented in the study are included in the article/Supplementary Material, further inquiries can be directed to the corresponding author.

## Author Contributions

SM, GQ, FF, and RS contributed to conception and design of the study. GQ performed the sampling collection. SM performed the laboratory treatment of the samples. GQ and SM performed the statistical analysis. All authors contributed to manuscript revision, read, and approved the submitted version.

## Conflict of Interest

The authors declare that the research was conducted in the absence of any commercial or financial relationships that could be construed as a potential conflict of interest.
