# Supplementary figures and images for: Characterization of Stormwater Runoff Based on Microbial Source Tracking Methods
Source: Front Microbiol. 2021 Jun 10;12:674047. doi: 10.3389/fmicb.2021.674047 (PMC8222924; doi:10.3389/fmicb.2021.674047)

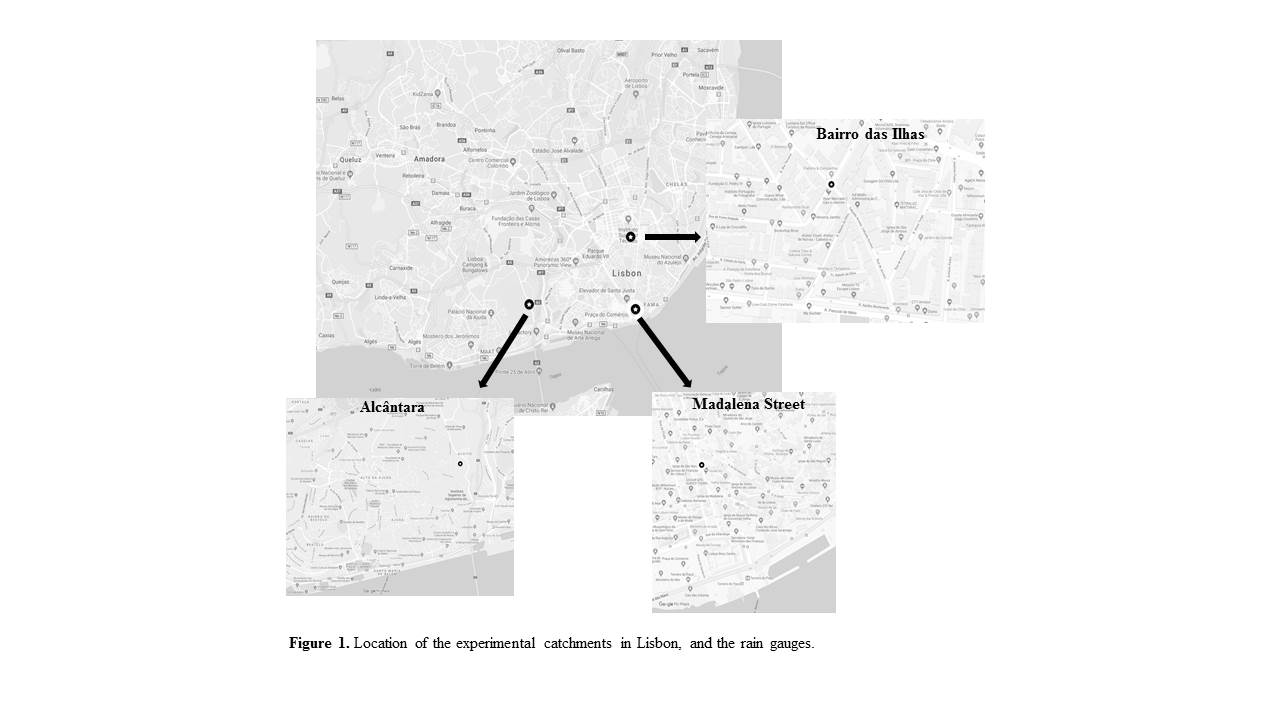

Supplement: Supplementary file 1 [file Image_1.jpeg]

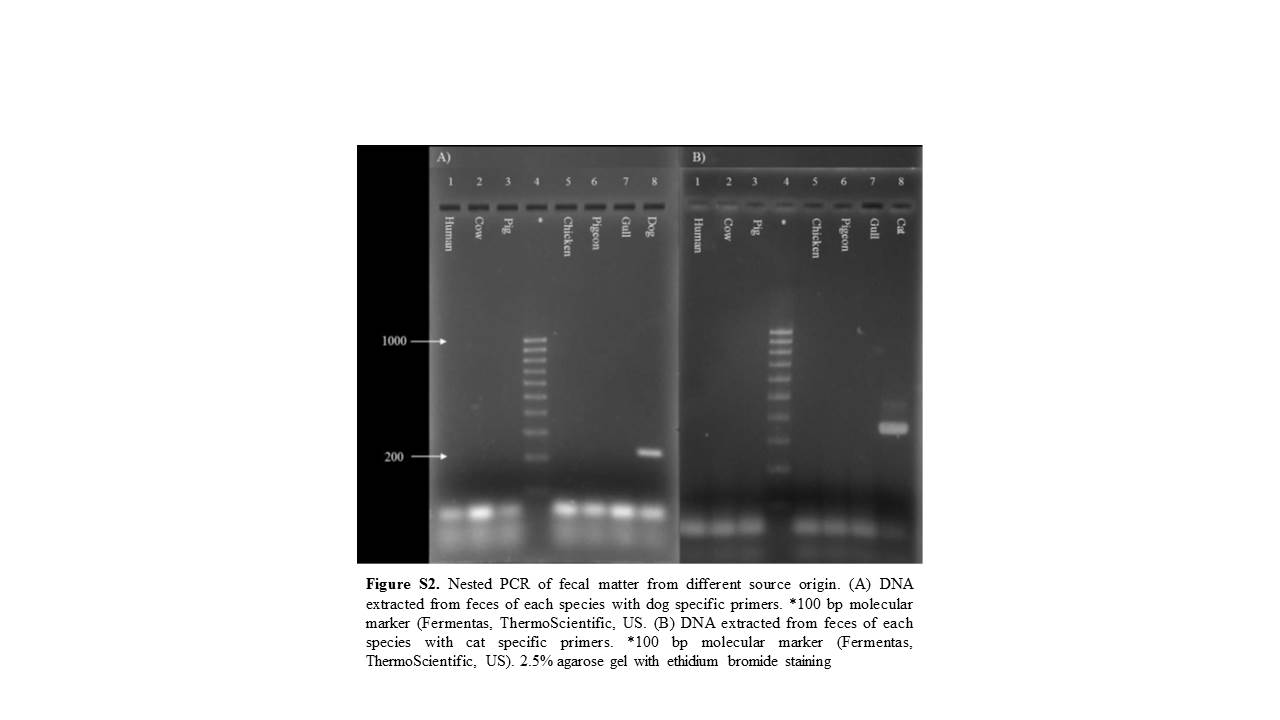

Supplement: Supplementary file 2 [file Image_2.jpeg]

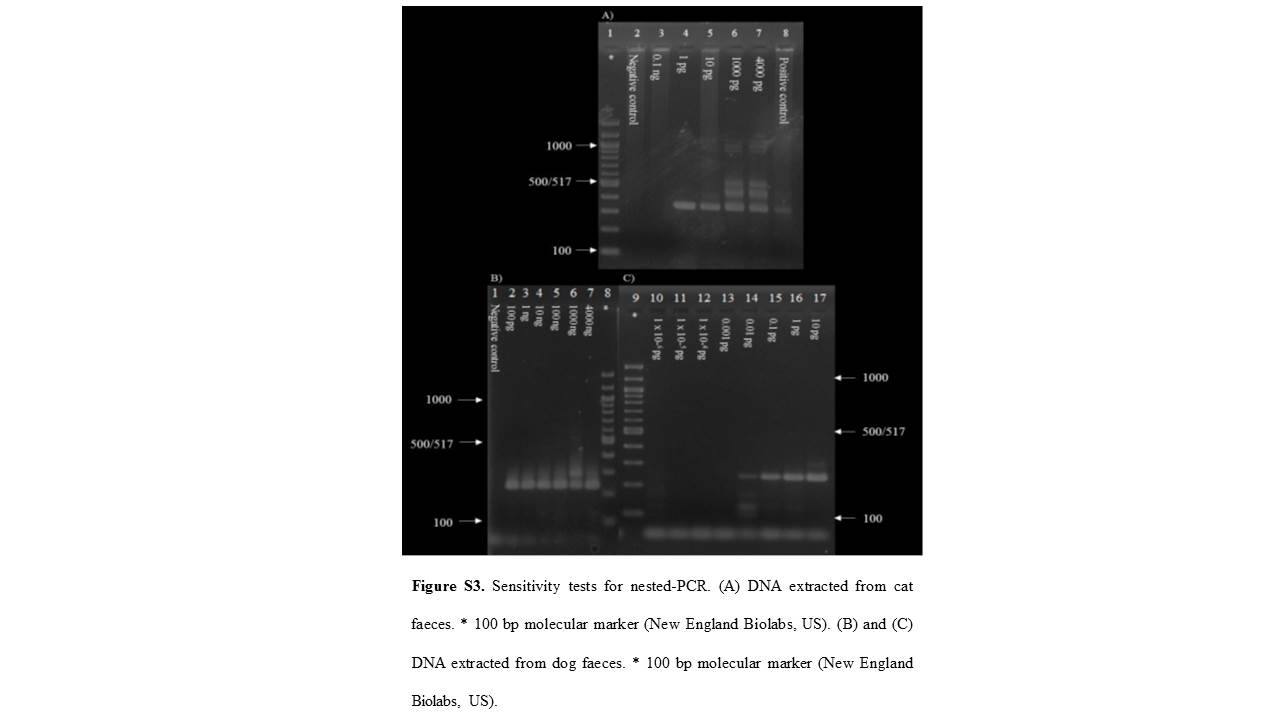

Supplement: Supplementary file 3 [file Image_3.jpeg]

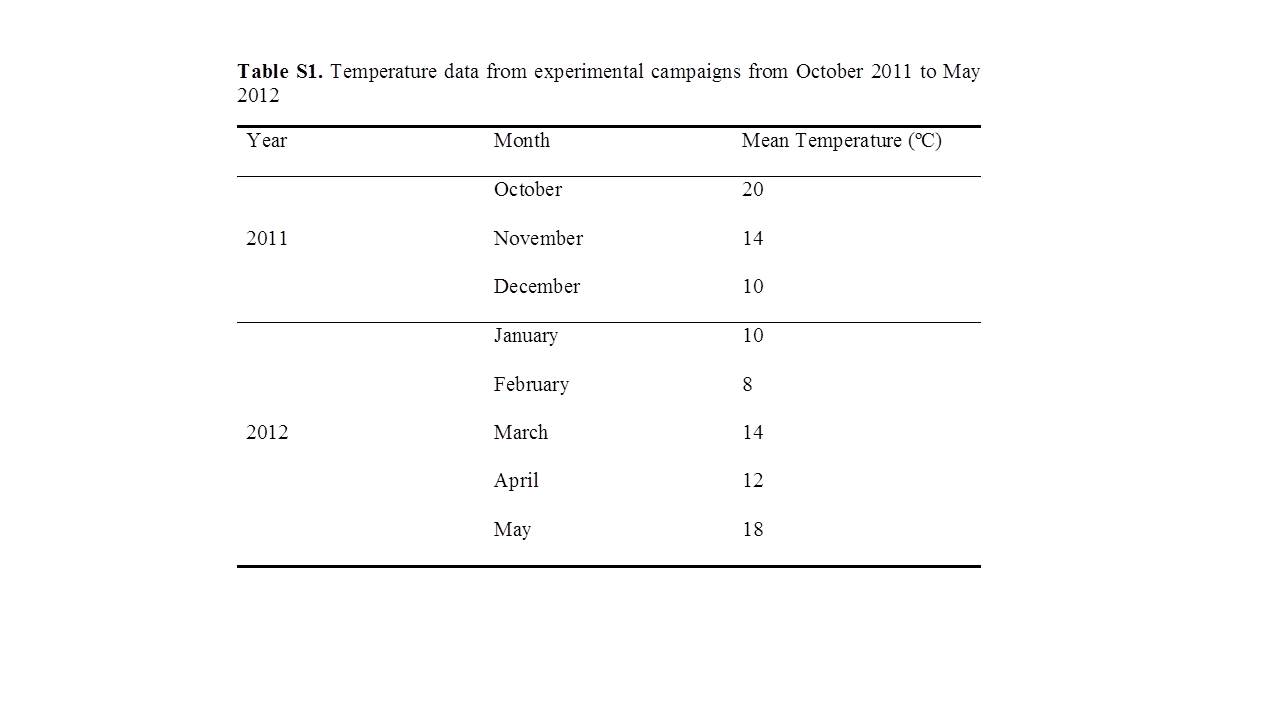

Supplement: Supplementary file 4 [file Image_4.jpeg]
